# Structural Rearrangements Maintain the Glycan Shield of an HIV-1 Envelope Trimer After the Loss of a Glycan

**DOI:** 10.1038/s41598-018-33390-2

**Published:** 2018-10-09

**Authors:** Roux-Cil Ferreira, Oliver C. Grant, Thandeka Moyo, Jeffrey R. Dorfman, Robert J. Woods, Simon A. Travers, Natasha T. Wood

**Affiliations:** 1 South African Medical Research Council Bioinformatics Unit, South African National Bioinformatics Institute, University of the Western Cape, Cape Town, South Africa; 2 Complex Carbohydrate Research Center, University of Georgia, Athens, Georgia United States; 3 Division of Immunology, Department of Pathology, University of Cape Town, Cape Town, South Africa; 4 Division of Immunology, School of Pathology, University of the Witwatersrand, Johannesburg, South Africa; 5 University of Cape Town, UCT Computational Biology Group, Department of Integrated Biomedical Sciences, Institute of Infectious Disease and Molecular Medicine, Cape Town, South Africa

## Abstract

The HIV-1 envelope (Env) glycoprotein is the primary target of the humoral immune response and a critical vaccine candidate. However, Env is densely glycosylated and thereby substantially protected from neutralisation. Importantly, glycan N301 shields V3 loop and CD4 binding site epitopes from neutralising antibodies. Here, we use molecular dynamics techniques to evaluate the structural rearrangements that maintain the protective qualities of the glycan shield after the loss of glycan N301. We examined a naturally occurring subtype C isolate and its N301A mutant; the mutant not only remained protected against neutralising antibodies targeting underlying epitopes, but also exhibited an increased resistance to the VRC01 class of broadly neutralising antibodies. Analysis of this mutant revealed several glycans that were responsible, independently or through synergy, for the neutralisation resistance of the mutant. These data provide detailed insight into the glycan shield’s ability to compensate for the loss of a glycan, as well as the cascade of glycan movements on a protomer, starting at the point mutation, that affects the integrity of an antibody epitope located at the edge of the diminishing effect. These results present key, previously overlooked, considerations for HIV-1 Env glycan research and related vaccine studies.

## Introduction

A key scientific challenge in the field of HIV-1 vaccine development is the design of immunogens that elicit antibodies capable of neutralising the wide range of HIV-1 isolates in circulation, despite its immense global diversity^1^. Due to immune-mediated selection pressure, the majority of this diversity is in the viral *envelope* gene that encodes the Env proteins on the surface of a virion^2^. The Env proteins facilitate viral entry to target cells and are formed by gp120/gp41 heterodimers that non-covalently associate, forming a trimer of heterodimers^3,4^. Even though the majority of HIV-infected individuals mount an immune response targeting these Env trimers, within-host diversity ensures that certain strains continue to evade recognition and neutralisation^5–8^.

Large sections of the Env trimers are covered by dense glycosylation and roughly half of its molecular mass is made up by glycans^9,10^, which have been suggested to protect the virus from antibody binding and neutralisation^11–15^. Changes in these glycosylation patterns can therefore have a large impact on its ability to escape from immune attack. Once HIV infiltrates the host cells, it takes advantage of host cellular biosynthetic pathways for its own benefit, which includes protein glycosylation as one of the main post-translational modifications^16^. N-linked glycosylation occurs in the endoplasmic reticulum and Golgi apparatus, where glycans are attached to asparagine residues within an Asn-X-Ser/Thr motif (X is any amino acid except proline^16^). The attached glycans, initially assumed to be immunologically inert “self” molecules, were until recently considered a largely insurmountable challenge for antibody recognition; hence termed, the “glycan shield”^8,17^.

However, some HIV-1 infected individuals develop potent and broadly neutralising antibodies (bNAbs) that specifically target, or find ways to bypass, the glycan shield^12,18–21^. These bNAbs are characterised by their target region, and are generally defined by particular monoclonal antibodies that target specific regions: the CD4 binding site^22^, the membrane proximal external region of gp41^23^, the glycan outer domain (typified by mAb 2G12)^24^, the V1V2 apex region around glycan N160^25^, the V3 base around glycans N301 and N332^26^, and the gp120/gp41 interface^27^. Despite the presence of such bNAbs in the serum of infected individuals, circulating plasma viruses generally escape, resulting in continued infection^28,29^. This escape from bNAbs has been linked to shifting glycosylation sites or mutations in the protein sequence surrounding specific glycans^11,12^. For example, Lynch *et al*.^11^ showed that the mutation introducing a glycan at position N276 lead to partial VRC01 resistance, and Moore *et al*.^12^ reported that the shift of glycan N332 to position N334 resulted in PGT128 resistance.

To quantify the impact of glycans on the effectiveness of neutralising antibodies, previous *in vitro* studies have used targeted de-glycosylation to compare the neutralisation of a range of viral strains with and without a specific glycan^13–15,30^. For example, the removal of glycan N301 (HXB2 numbering throughout), which is highly conserved^31,32^ amongst HIV strains, has been shown to expose V3 loop and CD4 binding site epitopes^33–37^. However, Moyo *et al*.^13^ described a subtype C strain, CAP45.2.00.G3 (referred to herein as CAP45.G3), in which removal of the glycan at position 301 unexpectedly did not result in increased sensitivity to to a large proportion of sera (61/64 panels) from chronically infected individuals^13^, despite its central role in protecting other isolates from neutralisation^13,14,18,33,34,36,37^. Furthermore, the N301A mutant had increased resistance to the CD4 binding site bNAb VRC01, and other VRC01-like bNAbs, when the neutralisation profile was compared to that of the wild-type^13^. The authors suggested that this virus typified a subset of viruses that could tolerate the loss of glycan N301 while largely maintaining the protective qualities of the glycan shield. The mechanism of compensation for the loss of a glycan, in this case glycan N301, as well as the development of increased resistance to VRC01, was not understood.

Here, we explored glycan conformations *in silico* to explain these findings. We analysed two molecular dynamics simulations of glycosylated Env trimers: the CAP45.G3 wild-type and the CAP45.G3 N301A mutant, which removes the glycosylation site at residue 301, to establish whether the models replicated the *in vitro* compensation of the glycan shield observed previously^13^. Subsequently we describe, in detail, the structural changes of glycans N442, N446 and N262 that bear the burden of compensation, how this burden is distributed, and the differences observed between the protomers of each model. We show that by determining the glycan nearest to each protein residue over time, we can clearly illustrate how changes in glycan conformations impact their ability to protect certain residues of the underlying protein. Finally, our study demonstrates how a cascade of events could contribute towards the increased resistance to antibodies targeting an epitope distal to the point mutation, in this case the N301A mutation and the VRC01 epitope. These *in silico* data provide a detailed investigation of the glycan shield’s ability to compensate for the loss of a glycan as well as the associated cascade of events that affect a distal epitope, which provides further important considerations and avenues of exploration for vaccine studies focussing on the HIV-1 Envelope.

## Results

**Figure 1:**
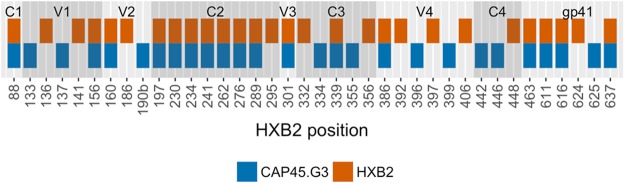
Distribution of potential N-linked glycosylation sites (PNGSs) for the CAP45.G3 strain (blue) relative to the PNGSs of the HIV-1 reference strain, HXB2 (orange). The gp160 conserved (C1-C4), variable (V1-V5) and gp41 sequence regions are labelled and shaded to indicate the borders of each region.

**Figure 2:**
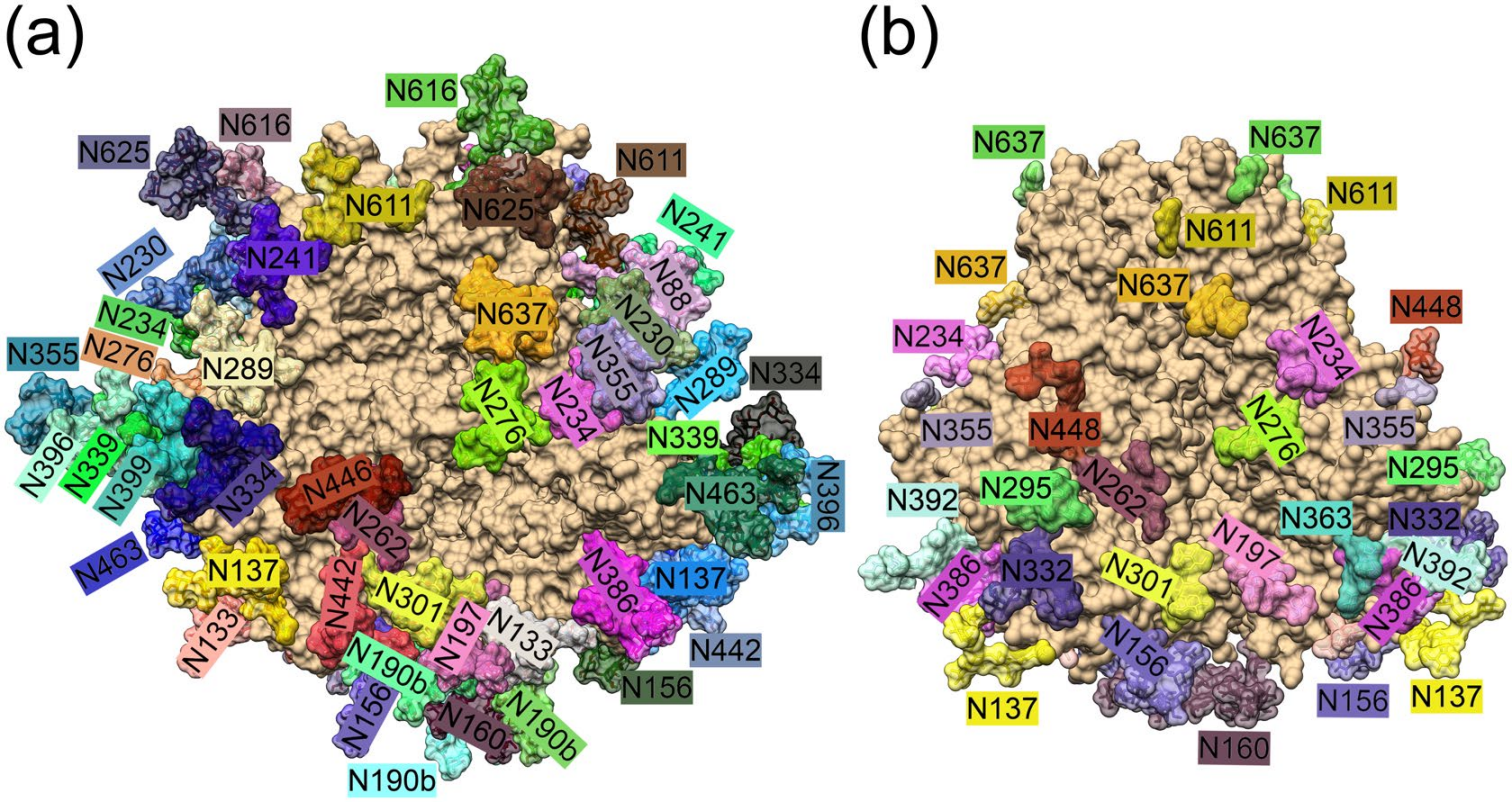
3D representation of the N-linked glycosylation sites of the (**a**) CAP45.G3 strain (computationally determined) and the clade A BG505 strain^58^ (crystal structure). The protein and glycan residues are shown as surfaces and the glycans are labelled according to HXB2 numbering. The depicted orientation is such that the lipid membrane is located at the top and the V1/V2-loop regions are at the bottom of the figure.

The CAP45.G3 subtype C virus was used to model the wild-type and N301A mutant trimer structures, comprising of protomers A, B and C, for molecular dynamics (MD) simulations. The CAP45.G3 Env sequence has 29 potential N-linked glycosylation sites (PNGSs, Fig. 1) and although we could not computationally glycosylate each PNGS, given the extent of variation seen during glycan occupancy studies^38–50^, the generated model represents one possible form of the wild-type glycosylated model (Fig. 2). The N301A mutant model matched the wild-type model, except the asparagine at position 301 was replaced with alanine and the glycan excluded. The systems were equilibrated for the first 20 ns of the simulation and analyses were performed on the remaining 500 ns.

### Region-specific changes in the average antibody-accessible surface area (AASA) between the wild-type and N301A mutant viruses

To investigate how the glycan shield compensates for the loss of glycan N301 in CAP45.G3, we calculated the antibody accessible surface area (AASA) of the wild-type and N301A mutant models using a 10 Å probe (as an approximation of the size of an antibody^51^) with Naccess^52^. When directly comparing AASA values between the wild-type and mutant simulations, there are differences due to underlying protein movement (particularly in the loop regions) and as a result of the loss of a glycan. In order to isolate the effect of the glycan loss, we divided the AASA of each residue by its AASA when all glycans are removed from the same simulation. The non-glycosylated value represents the “maximum” possible AASA for a residue. We next compared these AASA ratios between the wild-type and N301A mutant simulations using a bootstrap approach (see Methods section) to calculate which residues displayed a statistically significant (5% significance level) increase in the average AASA ratios between the wild-type and N301A mutant simulations.

**Figure 3:**
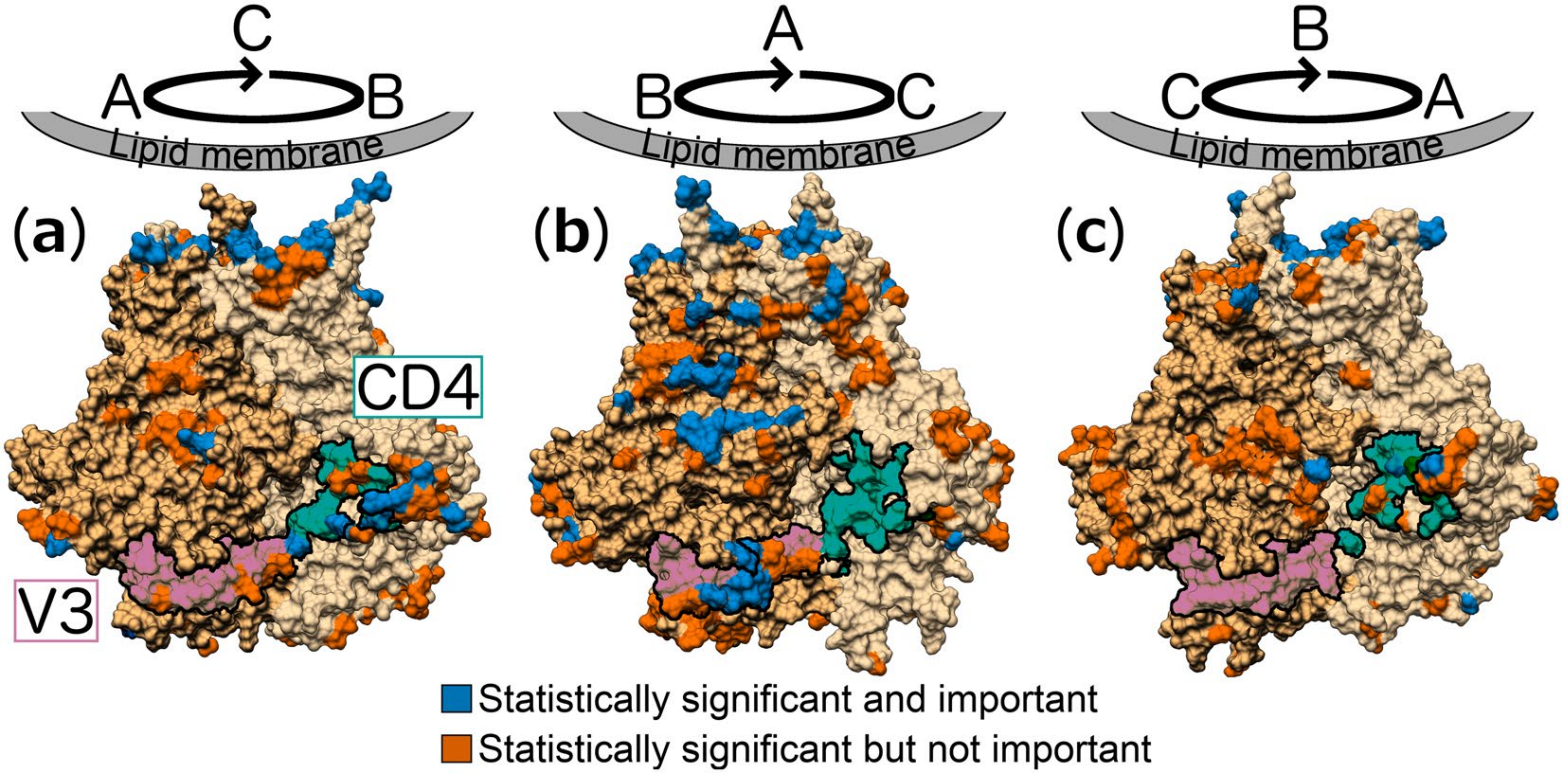
N301A mutant model with different orientations (**a–c**, 120° increments) to show each protomer (different shades of tan) and the residues with statistically significant increases in their average AASA ratios relative to the wild-type simulation (difference in average AASA <10% is orange (not important), ≥10% is blue (important)). A statistically significant increase was evaluated at a 5% significance level using a bootstrap approach (see Methods). The V3 (pink) and CD4 (green) regions are outlined. The depicted orientation is such that the lipid membrane is located at the top and the V1/V2-loop regions are at the bottom of the figure.

From the statistically significant subset (Fig. 3, blue and orange residues), we distinguished those residues with a considerable increase in their AASA ratios, i.e. an average increase of 10% or greater, which we define as ‘substantial’ (Fig. 3, blue residues), to further isolate those residues that were most affected by the loss of glycan N301. Residues identified in this manner were observed across all protomers and a total of 14, 42 and 18 residues were found for protomers A, B and C, respectively. Two clusters, one within the V3 region (Fig. 3, pink and outlined) and one within the CD4 binding site region (Fig. 3, green and outlined), were apparent.

These clusters (Fig. 3(a,b), blue clusters) with a substantial increase in their AASA ratios (average increase of 10% or greater) surround the N301A mutant site, and since the clusters fall within the V3 loop and CD4 binding site regions, we defined each cluster as the “V3 sub-region” (including residues 303–305, 321–322, 323, and 440) and “CD4 sub-region” (including residues 198, 365, 367, 368, 460, 462, and 464) (Table 1).

**Table 1:** Residues with significantly different AASA values between the wild-type and N301A mutant simulations.

Sub-region	[Env region] residues
V3	[V3] 303–305, 321–322, 323, [C4] 440
CD4	[C2] 198, [C3] 365, 367, 368, [V5] 460, 462, 464

For these V3 and CD4 sub-regions, we calculated the average AASA ratios over time (glycosylated region total, over non-glycosylated total averaged over time) for the protomers of the wild-type and N301A mutant models (Table 2). Apart from the CD4 sub-region on protomer A, the average AASA values are substantially higher for the N301A mutant sub-regions on protomer B (33% for the CD4 sub-region and 14% for the V3 sub-region, N301A mutant virus) than for the other N301A mutant or wild-type protomers.

**Table 2:** Average antibody accessible surface area (AASA) ratios (%) of the residues for the wild-type (WT) and N301A mutant (M) viruses that form part of the CD4 sub-region and V3 sub-region.

	Protomers					
	A		B		C	
	WT	M	WT	M	WT	M
CD4 sub-region	30	30	8	33	20	4
V3 sub-region	<1	0	<1	14	<1	<1

Because the laboratory study showed that the N301A mutant strain had increased resistance to VRC01, and VRC01-like, antibodies compared to the wild-type strain, we also calculated the AASA ratios for the residues that form part of this epitope (residues [C1] 123, 128, 129, [C2] 276, 278–283, [C3] 365–368, 371, [C4] 427–430, 455–459, [V5] 460–461, 463, 465–467, 469, 471, [C5] 472, 474, 476^53^). Apart from protomer C, where there was a substantial decrease in the AASA ratio for the N301A mutant model (wild-type: 11% and N301A mutant: 2%), the wild-type and N301A mutant ratios remained relatively unchanged for protomers A (wild-type: 11% and N301A mutant: 16%) and B (wild-type: 10% and N301A mutant: 14%).

### Glycan conformational changes around the N301A mutation

As shown in previous studies^11–15^, and evident from the AASA ratio results focussing on glycan N301, glycosylation shields the underlying protein. To fully understand the shielding capacity of any glycan, it is important to establish which glycans are capable, and most likely given their proximity, to affect the AASA of a particular protein residue. To achieve this, we determined the nearest glycan to each protein residue by calculating the distances between its atoms and all the atoms of each of the glycans for the wild-type and N301 mutant trimer models. Since the systems are dynamic, the nearest glycan can vary between frames and we defined each glycan’s ‘neighbourhood’ as the region that includes all the protein residues nearest to that glycan in the majority of the frames. For example, the protein residues surrounding glycan N442 form the neighbourhood of glycan N442 and can be represented on the 3D structure. These calculations allow us to determine which glycans shield the residues of the sub-regions identified during the AASA calculations (Table 1).

We specifically focus on comparing the neighbourhoods of the N301A mutant protomers to their wild-type counterparts, since the starting structures are identical and thus the differences in glycan neighbourhoods are likely to be due to the loss of glycan N301. To further visualise and interpret the 3D representation of the glycan neighbourhoods, we also calculate the average centre of mass position (to illustrate the directionality of the change), throughout the simulation, for each glycan. The results are described for the two sub-regions surrounding the N301A mutation, CD4 and V3, identified during the AASA calculations.

#### CD4 sub-region

**Figure 4:**
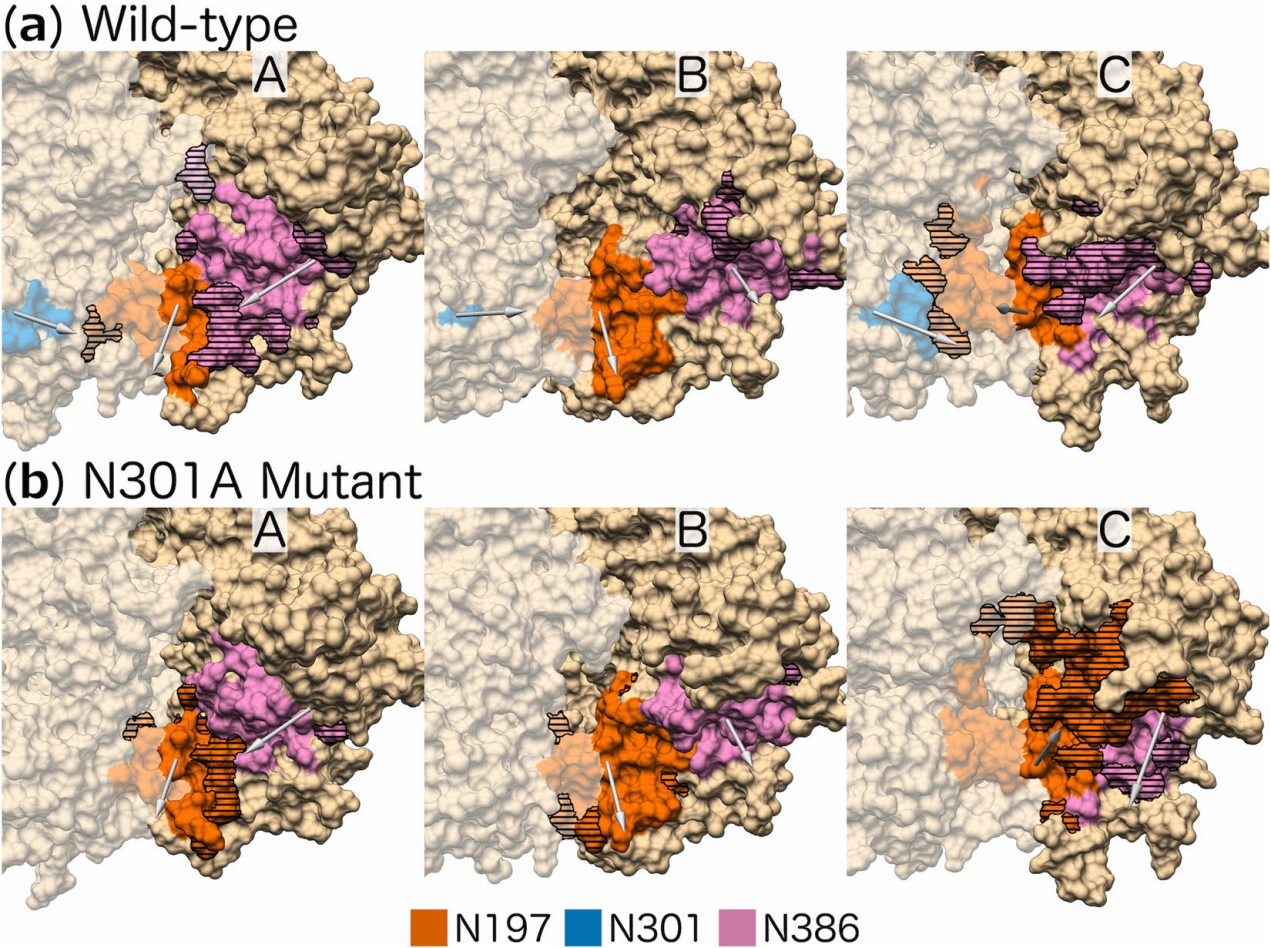
Neighbourhoods of the (**a**) wild-type and (**b**) N301A mutant glycans nearest to the CD4 sub-region for the three protomers (A, B, and C): glycans N197 (orange), N301 (blue, only present in the wild-type), and N386 (pink). Horizontal lines represent residues that are in different neighbourhoods when comparing the wild-type and N301A mutant. The arrows originate from the Cα atom of the Asn and end at the average centre of mass position for the selected glycans. The representations are cropped around the CD4 binding site of each protomer and the surface representation of residues on adjacent protomers are shown as opaque.

Our neighbourhood calculation determined which glycans were nearest to each of the residues within each sub-region. For the C3 residues (CD4 sub-region, Table 1), glycan N386 was the nearest to these residues for all wild-type protomers, as well as for protomers A and B of the N301A mutant, whereas glycan N197 was the nearest on protomer C of the N301A mutant (Table 3). To further investigate how glycan N197 replaces glycan N386 as nearest glycan to the C3 residues, we represent their 3D neighbourhoods on all protomers (Fig. 4). There is a clear difference in the conformation of glycan N197 (protomer C; N301A mutant) when compared to its wild-type counterpart, and this conformational change results in a shift in the residues contained in its neighbourhood (Fig. 4(a,b), protomer C). A large proportion (29%) of the neighbourhood of the wild-type glycan N197 (protomer C) contains residues from the V3 region of protomer B. However, the neighbourhood of glycan N197 of the N301A mutant includes a large proportion (31%) of CD4 binding residues from protomer C and five fewer V3 residues of protomer B than the wild-type.

**Table 3:** The glycans nearest to the two protein clusters, with a statistically significant and substantial (≥10%) increase in their average AASA ratio (CD4 and V3 sub-region), for the three protomers (A, B and C).

	Protomers		
	A	B	C
**CD4 sub-region:**			
Wild-type	N386, N463	N386, N355	N276, N386, N463
N301A mutant	N276, N386	N386, N463	N197, N276, N463
**V3 sub-region:**			
Wild-type	N156, N262, N442	N156, N197, N301, N442	N156, N197, N262, N301
N301A mutant	N156, N262, N442	N156, N442	N156, N262

Also, for protomer C of the N301A mutant, a change in the conformation of glycan N386 is associated with the change in conformation of glycan N197 (Fig. 4(b) protomer C), which causes the exclusion of any CD4 binding residues from glycan N386’s neighbourhood. Conversely, glycan N386 of the N301A mutant protomer A also undergoes a conformational change (Fig. 4(b) protomer A), but this change results in the inclusion of an equivalent number of CD4 binding residues as its wild-type counterpart.

#### V3 sub-region

**Figure 5:**
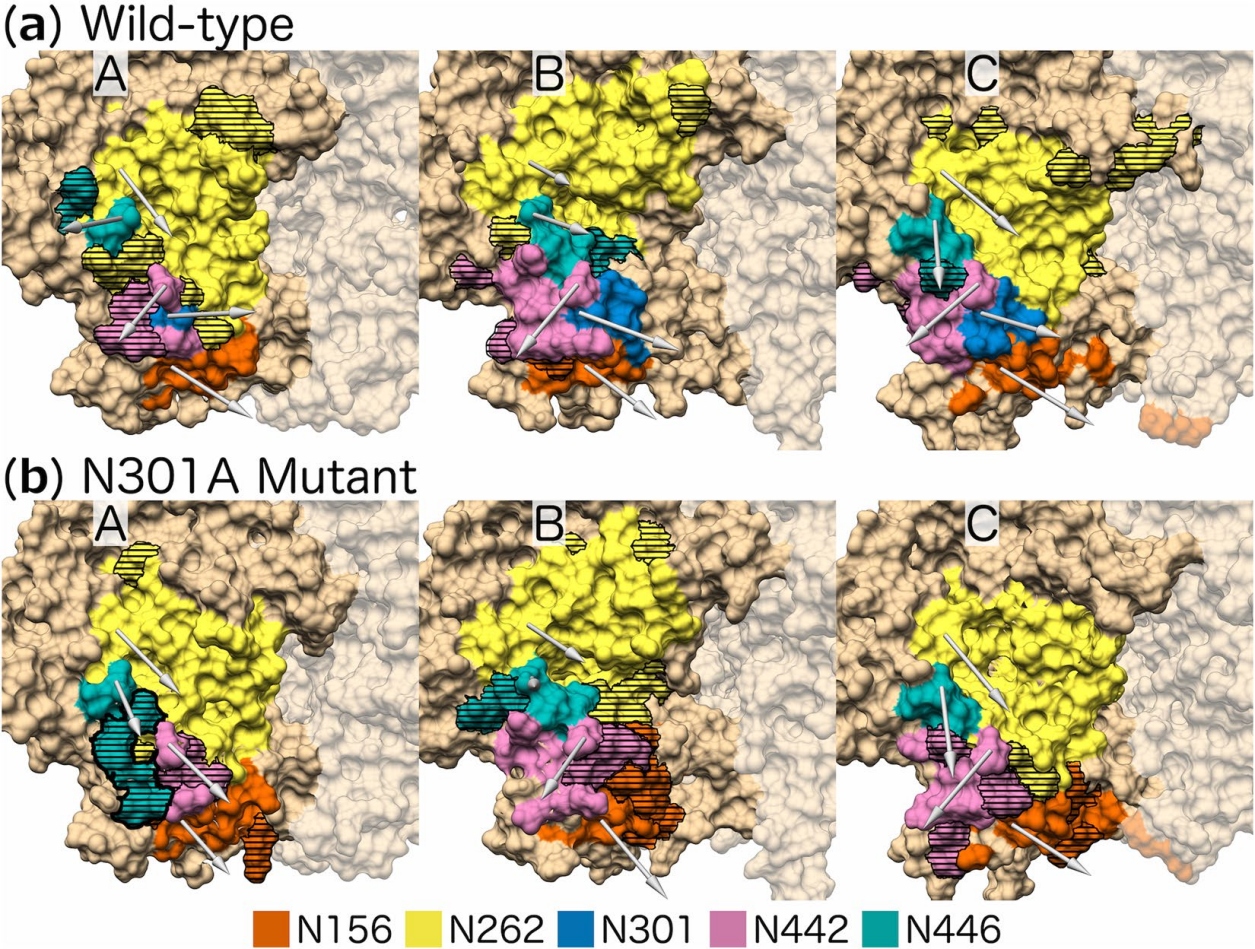
Neighbourhoods of the (**a**) wild-type and (**b**) N301A mutant glycans nearest to the V3 sub-region for the three protomers (A, B, C): glycans N156 (orange), N262 (yellow), N301 (blue, only present in the wild-type), N442 (pink), and N446 (green). Horizontal lines represent residues that are in different neighbourhoods when comparing the wild-type and N301A mutant. The arrows originate from the Cα atom of the Asn and end at the average centre of mass position for the selected glycans. The representations are cropped around the V3 region of each protomer and the surface representation of residues on adjacent protomers are shown as opaque.

The neighbourhoods of the glycans nearest to the V3 sub-region, N156, N262 and N442 (Table 3), are shown in Fig. 5. Additionally, the neighbourhood of glycan N446 is included because its neighbourhood is itself enclosed by the abovementioned V3 sub-region glycan neighbourhoods (Fig. 5(a) protomer B). There are marked differences between the conformations of glycans N442 and N446 of the N301A mutant model on protomer A, and to a lesser extent those on protomers B and C, when compared to their wild-type counterparts (Fig. 5).

Glycan N442 (N301A mutant) re-orientates towards the N301A site in all the protomers to varying degrees, with the largest shift occurring on protomer A (Fig. 5(b) protomer A) and only a slight movement on protomer C (Fig. 5(b) protomer C). Comparatively, glycan N446 (N301A mutant) re-orientates towards the N301A site on protomers A and C only (Fig. 5(b) protomers A and C), while for protomer B, the glycan shifts away from the N301A site (Fig. 5(b) protomer B). Finally, glycan N262 on protomer C (N301A mutant) also re-orientates towards the N301A site (Fig. 5(b) protomer C).

### Protomer-specific glycan conformational changes that compensate for the loss of glycan N301

Since glycans N197, N262, N386, N442 and N446 were identified as the glycans nearest to the protein residues that had increased AASA ratios and showed conformational changes, we investigated whether these conformations were viable when glycan N301 was present, i.e. whether or not they caused large conformational clashes with any of the wild-type glycans, which would imply that the N301A mutant glycan conformations are impossible in the wild-type model. Hence, we superimposed the N301A mutant glycan conformations of each protomer onto its wild-type counterpart and directly compared each time point. For each of these N301A mutant glycans, we calculated the proportion of frames where an atomic overlap was observed with any of the wild-type glycans, as well as the average number of atoms with an atomic overlap with wild-type glycans (Table 4). Due to differences between the results, each protomer is described separately.

**Table 4:** Overlap calculations between the N301A mutant glycan substitute and any wild-type glycan.

N301A mutant glycan substitute	Wild-type glycans that clash with the N301A substitute	Protomers		
		A	B	C
N197	N133		53%^a^; 45^b^	
	**N156** ^**c**^	**86%; 58**	**68%; 46**	**<1%; 2**
	N160		33%; 30	
	**N301**	**21%; 37**	**10%; 25**	**12%; 27**
	N386	55%; 25	<1%; 6	94%; 115
N262	N301	77%; 22	3%; 7	97%; 58
	N334		<1%; 1	16%; 6
	N442	90%; 42	12%; 13	67%; 26
	N446	6%; 9	99%; 175	85%; 23
N386	N137	9%; 5	17%; 67	
	N197	11%; 13	1%; 14	6%; 7
	N463	13%; 33	22%; 20	8%; 43
N442	N133			34%; 14
	N137		13%; 49	59%; 43
	N156	21%; 33	7%; 43	
	N262	52%; 9	2%; 13	1%; 6
	N301	99%; 226	29%; 28	19%; 14
	N446		31%; 45	36%; 19
N446	N137	15%; 13		1%; 12
	N262	>99%; 80	88%; 86	24%; 9
	N301	22%; 18	<1%; 16	66%; 36
	N334		24%; 56	3%; 49
	N442	95%; 148	6%; 16	96%; 125

^a^The proportion of frames with a clash (%).

^b^The average number of overlapping atoms in those frames with a clash. Wild-type glycans are omitted if the proportion of frames with an overlap is below 10% on all protomers.

^c^Wild-type glycans on a different protomer from that of the N301A mutant glycan are shown in bold.

#### Protomer A

The glycan with the largest and most frequent overlap with glycan N301 (protomer A; wild-type) was glycan N442 (N301A mutant). In 99% of the frames, glycan N442 occupies the same space as glycan N301 does in the wild-type simulation with an average of 226 overlapping atoms per frame (Table 4). A Man-9 glycan (described in the Methods section) has a total of 244 atoms; 226 clashes therefore imply that almost all of the atoms overlap with glycan N301 and that glycan N442 (N301A mutant) occupies most of the space vacated by glycan N301 for practically the entire duration of the trajectory. Similarly, glycan N262 (protomer C; N301A mutant) occupies some (22 clashes per frame) of the space vacated by glycan N301 for a large (77%) proportion of the trajectory (Table 4).

Concurrently, the space vacated by glycans N442 and N262, is in turn occupied by glycan N446 (N301A mutant). This is evident from the large proportion of frames with an atomic overlap, as well as a large average number of overlapping atoms per frame (Table 4), observed between glycan N446 and glycans N442 (95%, 148 overlapping atoms) and N262 (>99%, 80 overlapping atoms).

#### Protomer B

None of the glycans on protomer B have noticeably large overlap with glycan N301 on the wild-type protomer B, which suggests that the wild-type glycan conformations are maintained on the N301A mutant protomer B.

#### Protomer C

The largest overlap with glycan N301 were observed for glycan N262 (N301A mutant), where 97% of the frames have an atomic overlap with glycan N301, with an average of 58 overlapping atoms per frame, and for glycan N446 (N301A mutant; 66%, 36 overlapping atoms; Table 4).

Lastly, the nearest glycan results highlighted the conformational change of glycan N197 (protomer C; N301A mutant). This shifted conformation appeared to occupy the space where glycan N386 of the wild-type is located, which is confirmed here by the large overlap observed between glycan N197 (N301A mutant) and glycan N386 (wild-type). Only a small overlap is observed for glycan N386 (protomer C; N301A mutant) with glycan N301, which suggests that the adjusted conformation for this glycan is viable in the presence of glycan N301. The conformation of glycan N197 (protomer C; N301A mutant), however, has moderate overlap with glycan N301 (12%, 27 overlapping atoms). Further investigation revealed that glycan N197 overlaps with glycan N301 at two specific time intervals (14–35 ns and 346–500 ns) and not throughout the simulations. This suggests the ‘new’ conformation for glycan N197 can coexist with glycan N301.

### Domino effect of glycan conformational changes: how changes propagate and taper off

**Figure 6:**
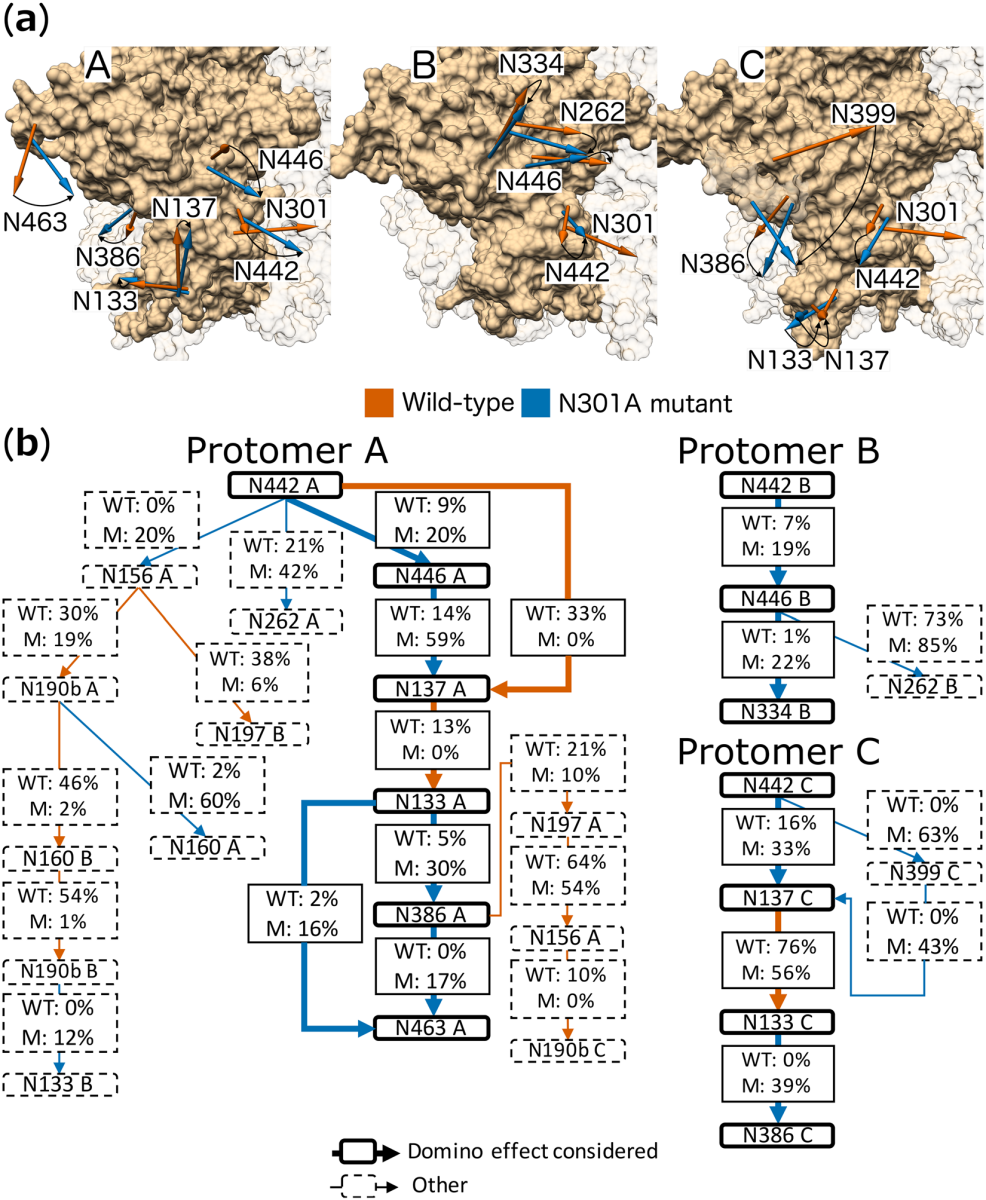
Domino effect of glycan conformational changes on the three protomers. (**a**) Structures illustrating the location of glycans. The arrows originate from the Cα atom of the Asn and end at the average centre of mass position for the selected wild-type (orange) and N301A mutant (blue) glycans identified by the hydrogen bond analysis for protomers A, B and C. Curved arrows indicate the degree of change in directionality, e.g. the large movement for glycan N399 on protomer C. The representations are cropped around the V3 region of each protomer and the surface representation of residues on adjacent protomers are shown as opaque. (**b**) Each schematic - protomer A, B and C - shows the hydrogen bond network starting from glycan N442 (first node) on protomer A, B and C, respectively. Solid borders around glycan nodes and broad arrows represent glycans and interactions that form part of the domino effect considered, whereas dotted borders and fine arrows indicate any glycans and interactions outside the considered domino effect. The arrows connecting glycan nodes distinguish interactions that are either greater on the wild-type (WT; orange) or on the N301A mutant (M; blue).

During the investigation of the protomer specific conformational changes, we observed that the space generated by removing glycan N301 is occupied by another glycan, which in turn leaves a new space that is then occupied by yet another glycan. To extend the investigation of glycan conformational changes beyond those glycans nearest to the N301A mutation site, while maintaining the relevance of the changes (i.e. the changes associated with the loss of the N301 glycan), we compare the frequency of the hydrogen bonds that form between glycans in the wild-type and N301A mutant. Substantial changes in the hydrogen bond network allude to changes in the conformations of glycans adjacent to those glycans near the N301A mutation site. For each of the identified glycans, we calculated the average centre of mass position, throughout the simulation, for the wild-type (Fig. 6(a), orange arrows) and N301A mutant (Fig. 6(a), blue arrows).

#### Protomer A

The conformational change domino effect is clear on protomer A, where the conformations of six glycans (N442, N446, N137, N133, N386 and N463) are affected. In the wild-type, these glycans are interlinked by sequential hydrogen bonds. However, the interlinked nature of these glycans is broken in the absence of glycan N301, and two glycan clusters exist on the N301A mutant. The first cluster includes glycans N442, N446 and N137, and the second glycans N133, N386 and N463.

Glycan N442 occupies the space vacated by glycan N301 (Fig. 6(a) protomer A). In turn, the space vacated by glycan N442 is occupied by glycans N446 and N137 (Fig. 6(a) protomer A). These rearrangements result in an increased interaction between glycans N442 and N446 (9% wild-type vs. 20% N301A mutant; Fig. 6(b) Protomer A). Glycan N137 on the other hand, no longer interacts with glycan N442 (33% wild-type vs. 0% N301A mutant; Fig. 6(b) Protomer A) but instead has an increased interaction with glycan N446 (14% wild-type vs. 59% N301A mutant; Fig. 6(b) Protomer A). The second cluster is separated from the first by the change in conformation of glycan N137 (Fig. 6(a) protomer A), which eliminates the interaction between glycans N137 and N133 (13% wild-type vs. 0% N301A mutant; Fig. 6(b) Protomer A). This reduced structural hindrance near glycan N133 may allow for its re-orientation (Fig. 6(a) protomer A) and subsequent increased interaction with glycan N386 (5% wild-type vs. 30% N301A mutant; Fig. 6(b) Protomer A), as well as with glycans N463 (2% wild-type vs. 16% N301A mutant; Fig. 6(b) Protomer A). There is also an increased interaction between glycan N386 and N463 (0% wild-type vs. 17% N301A mutant; Fig. 6(b) Protomer A). Other changes in the hydrogen bond network quickly taper off to glycans located at the protein apex (Fig. 6(b) Protomer A).

#### Protomer B

Apart from glycans N156, N197, N262, N386, N442 and N446, which were previously investigated on protomer B during the neighbourhood and subsequent overlap analyses, the hydrogen bond network further identifies interaction with glycan N334 (N301A mutant; Fig. 6(b) Protomer B). However, glycan N334 shows no movement when compared to its wild-type counterpart (Fig. 6(a) protomer B), which indicates that there was almost no domino effect.

#### Protomer C

The domino effect was similar to that of protomer A, however, glycans N446 and N463 (N301A mutant) are not implicated, whereas glycan N399 is. The hydrogen bonds between glycans N137 and N133 persist, although at a reduced scale compared to the wild-type (76% wild-type vs. 56% N301A mutant; Fig. 6(b) Protomer C). The hydrogen bonds between glycans N133 and N386 were not present in the wild-type and are substantial in the N301A mutant (0% wild-type vs. 39% N301A mutant; Fig. 6(b) Protomer C). The persistent interaction between glycans N133 and N137 results in all these interlinked glycans collectively moving closer to the gap left by the N301A mutation on the N301A mutant model (Fig. 6(a) protomer C). In our model, this movement, coupled with the increase in interaction between glycans N133 and N386, likely contributes to the ‘new’ conformation of glycan N197 (protomer C; N301A mutant).

## Discussion

Here, we analysed two 500 ns molecular dynamics simulations (CAP45.G3 wild-type and CAP45.G3 N301A mutant) and show that the systems imitate *in vitro* neutralisation data – the glycan shield restores itself and retains its ability to protect key epitopes after the removal of glycan N301^13^. This was in contrast to a second isolate, Du156.12, where the laboratory results showed that this N301A mutant virus had increased sensitivity to a panel of sera from chronically infected individuals^13^, and where the *in silico* simulations of the Du156.12 wild-type and N301A mutant models were vastly different to that of the CAP45.G3 models (Supplementary File 1). The key observation during our initial, *in silico*, comparative analysis was that the conformational differences of the glycans on the wild-type models, as well as the landscapes around each of these glycans, likely affected the ultimate changes in the glycan shields. The importance of this collective behaviour of glycans on the Env surface was previously noted by Lemmin *et al*. describing several, relatively stable, glycan microdomains^54^. Since Moyo *et al*.^13^ speculated that the CAP45.G3 virus typified a subset of viruses where the loss of glycan N301 was tolerated, i.e. the protective qualities of the glycan shield, or perhaps the glycan microdomain, were retained, the focus of our manuscript was on providing a thorough account for the suggested compensation. Therefore, for the CAP45.G3 modelled systems, we describe, in detail, the change in the glycan landscape, and cascade of events, that may have contributed towards the maintenance of the glycan shield for this viral isolate. The only structural difference between the model simulations is the presence or absence of the N-linked glycan at position 301 and the stability of the distinct glycan interactions throughout each simulation suggests that the observed differences are meaningful.

We observed clear evidence of compensation for two of the three N301A mutant protomers (A and C) within the trimeric protein structures. The antibody accessible surface area (AASA) ratios of these two protomers are equivalent to their wild-type counterparts, despite the lack of glycan N301. However, unexpectedly, there are differences in the particular glycans compensating for the loss. Glycan N442 bears the bulk of the compensation burden on protomer A, whereas glycans N446 and N262 share the burden on protomer C. However, even though glycan N442 bears the compensation burden on protomer A, its ‘new’ conformation was considerably influenced by glycan N446 on the same protomer. The spatial pressure from glycan N446 resulted in a glycan N301-like conformation observed for glycan N442 (protomer A; N301A mutant). Therefore, this suggests that in our model, glycan N446 is essential for maintaining the glycan shield by not only directly bearing a part of the compensation burden, but also through its substantial influence on the conformation of glycan N442. Although we are currently unaware of studies describing the influence of glycan N446 on epitopes accessibility, there have been reports on glycan N442 and its role in shielding epitopes of the CD4 and gp41 regions^15^. This suggests that glycan N446 may also influence the availability of epitopes in these regions.

In contrast to protomers A and C, we found ambiguous evidence of compensation for protomer B (N301A mutant), where increased AASA ratios were observed for various residues. The apparent lack of compensation for protomer B can be, in part, attributed to the lack of conformational change in glycan N442, as well as the substantial change in the conformation of glycan N197 on protomer C (adjacent to protomer B), which is commonly reported as shielding the CD4 and V3 regions^14,15^.

We also identified specific glycan conformational changes that have a direct impact on the accessibility of the modelled protein surface. The conformational change of glycan N197 (protomer C; N301A mutant) has drastic implications for the AASA ratios of the residues of the CD4 sub-region and VRC01 epitope on protomer C. Both the residues in the CD4 sub-region (Table 1) and the residues of the VRC01 epitope show decreased AASA ratios for protomer C of the N301A mutant model. Glycan N386 (protomer A; N301 mutant) also plays a role in maintaining the AASA ratio of residues that form part of the CD4 sub-region (Table 1); however, in comparison, the conformational change of glycan N386 does not lead to a decrease in the AASA ratio of the VRC01 epitope, but instead, the removal of glycan N301 leads to a slight increase in AASA for this region (protomer A). Thus, based on the AASA results, glycan N386 may not contribute to the increased resistance observed to the VRC01 bNAb (unlike glycan N197), which is contrary to evidence of this glycan shielding the CD4 binding site^30,55^.

Despite the popularity of calculating the AASA^51,54,56,57^ as a predictor of whether residues are accessible by antibodies, it remains to be seen whether they are useful, and accurate, for describing the contact between HIV-1 gp160 and antibodies. For example, there are considerable differences between the conformation of particular HIV-1 envelope glycans co-crystalised with VRC01 compared to an unliganded crystal structure^58^. Additionally, Stewart-Jones *et al*.^58^ showed that substantial overlap occurred between broadly neutralising antibodies and one or more glycan/s throughout 500 ns trajectories, suggesting that known broadly neutralising antibodies likely need to accommodate at least one glycan during the binding process. Given this evidence, the passive nature of AASA calculations is likely to result in an underestimation of the accessibility of residues in regions where the “shielding” glycans can be flexible. This may be the case for glycan N386 (protomer A; N301A mutant), where the flexibility of glycan N133 (protomer A; N301A mutant) increased the structural hindrance near glycan N386. This, in turn, reduced the freedom of movement of glycan N386 resulting in slightly higher AASA ratios around this glycan in our model, but potentially still leading to increased VRC01 resistance due to its reduced flexibility to accommodate the antibody. However, complete resistance to VRC01 was not observed for the CAP45.G3 N301A mutant^13^ and it is unknown if glycan N386 plays a role, or whether the conformation of glycan N197 is solely responsible for, the observed increase in VRC01 resistance.

Due to the potential caveats associated with AASA, we extended the analyses to examine the capacity for each glycan to act as a shield to its surrounding protein residues by determining which glycan was the nearest to each protein residue across time. A benefit of this analysis is the ability to generate leads in the cases where the binding site of a particular antibody is known and point mutations that would reduce resistance are sought. The analysis also provides an intuitive and easy representation of the glycan positions over time. For example, the large change in the conformation of glycan N197 (protomer C; N301A mutant) is apparent and easily related to a change in the conformation of glycan N386 (protomer C; N301A mutant; Fig. 4(b)).

The nearest glycan approach also presents certain disadvantages; small differences are easily overlooked and, since the crowded nature of the HIV glycan shield can result in large conformational differences that change the glycan neighbourhood only slightly, small changes could be an important consideration. Additionally, the nearest glycan may not necessarily be the shielding glycan, which is the case when glycans are buried and therefore relatively inflexible. Glycan N262, where a large neighbourhood was observed, presents one such case. Visual inspection suggested that other glycans, only slightly further away, may have greater capacity to shield certain residues assigned to glycan N262. However, due to its static, buried, nature, these residues were consistently assigned to glycan N262, and therefore removing it prior to the analysis might be prudent.

The length of the trajectory (MD simulation), which relates to the uncertainty associated with observing enough of the sample space during the simulation is a key, and ongoing, problem of molecular dynamics research of large structures. We would expect that, on a long enough time scale, the glycans would populate similar volumes across the protomers. However, the glycans of each protomer adopt distinct conformations that do not interconvert during our simulation. Unlike the protomer scissoring reported by Lemmin *et al*.^54^, we did not observe this effect for either the wild-type or N301 mutant model simulations, but did identify small scale, protomer specific, protein movements (data not shown). Nonetheless, despite the differences, the data for each protomer is self-consistent and stable; i.e. although the effect of glycosylation on the AASA is different across protomers, the effect is constant in each protomer throughout the simulation. A possible solution to this issue is to use replicate exchange (which should explore greater proportions of the potential energy surface) but requires more study. Yang *et al*.^56^ used replicate exchange and described enhanced sampling for glycosylated Env trimers; it would be worthwhile for future studies to compare the degree of convergence in the observed glycan shapes between different protomers in the different approaches. The advantage of the molecular dynamic protocol used here is that it clearly shows that there are glycan-glycan and glycan-protein interaction networks that extend across the Env surface and persist on at least a 500 ns timescale. Although the lack of convergence between protomers is concerning for drawing statistically strong conclusions, it is interesting, and important, to note that the interactions driving the observed differences occur at longer time scales than 500 ns.

Finally, our modelling results suggest that the loss of a glycan, due to a point mutation, can result in a cascade of events on the same protomer (intra-protomer) that could contribute towards increased resistance to epitopes distal to the location of the initial sequence mutation causing the cascade of events – in this case the VRC01 epitope. We initially speculated that this epitope would be influenced directly by the N301A mutation via cross-protomer interactions (through the additional space created), since it has been shown that the N301 glycan overlaps with VRC01 on an Env crystal structure bound to this antibody^58^. However, here we present evidence for intra-protomer conformational rearrangements of specific glycans, which we believe contributed to the increased resistance of the CAP45.G3 N301A mutant to VRC01, and other VRC01-like, antibodies.

It is unlikely that the glycan shield of the CAP45.G3 isolate is unique in its ability to compensate for a loss of a glycan, however, the particular glycan distribution and clustering meant that the absence of glycan N301 was not crucial for the maintenance of its protective qualities as a whole. The further implication is that different viral Env glycoproteins will likely each have their own set of glycans that are, individually, either dispensable or indispensable in forming, and maintaining, the glycan shield. This argument extends to the asymmetrical effects seen across protomers; the degree of glycan conformity, both in terms of site occupancy and glycan type, between the three protomers of an Env trimer is currently unknown and largely unacknowledged in HIV-1 Env studies. It is completely plausible that the HIV-1 glycans of Env trimers vary across their protomers and that these differences affect the neutralisation efficiency of glycan-dependent antibodies in different ways. This ties in with the knowledge that antibodies are not always present on all three protomers, and that glycan heterogeneity is one likely cause of this finding^59,60^.

Despite the caveats associated with molecular dynamics simulations, as well as the immense potential variation in the Env glycan shields of HIV-1 isolates, this study provides a detailed investigation of how the loss of a single HIV-1 Env glycan does not result in a hole, but rather results in a cascade of events that may have led to the maintenance of the glycan shield and increased resistance to a broadly neutralising antibody observed for the viral isolate. Given the focus on Env glycans within HIV-1 vaccine research, and the importance of these glycans for bNAb binding, we hope the techniques and results presented here will encourage further in-depth consideration of the virus-specific glycan landscapes. Future investigations, both *in vitro* and *in silico*, focussing on different glycan point mutations and including systems composed of a variety of glycan forms, will demonstrate to what extent these results translate, and are predictive, across viral isolates and subtypes.

## Methods

### Structural modelling and molecular dynamics simulations

#### System preparation

We used MODELLER^61,62^ to generate the protomer structures. The CAP45.G3 (Genbank accession number DQ435682) sequence was used as the target, and three reference structures, PDB IDs 4NCO^63^, 4TVP^51^ and 2B4C^64^ were used as starting templates. The modelling was repeated ten times and models were ranked according to their DOPE scores^65^. The model with the lowest DOPE score was then selected, triplicated, and the three copies were aligned to the protomers of the 4NCO trimer structure to generate the trimeric model. Potential N-linked glycosylation sites (PNGSs) were determined by identifying the NXT/S motifs, where X is any amino acid except a proline. For each PNGS on the trimer homology model, we attempted to attach a Man9GlcNAc2 glycan (Manα1–2Manα1–6[Manα1–2Manα1–3]Manα1–6[Manα1–2Manα1–2Manα1–3]Manβ1–4GlcNAcβ1–4GlcNAcβ1-). The glycans were generated using the carbohydrate builder available at Glycam-Web^66^ and were attached using a prototype tool under development for the Glycam-Web suite of web tools that explores the most populated rotamers of the Asn-GlcNac linkage^67^ and then, if clashes are observed between previously added glycans or the protein, iteratively rotates the Asn sidechain and glycosidic linkages within reasonable bounds. Two of the glycosylation sites, N335 and N678, were not glycosylated; site N335 occurs in an NNST motif and we were specifically interested in the positioning of glycan N334 in the context of bNAbs^12^ and due to its relative abundance compared to N335^32^, and N678 falls outside the modelled domain. For the remaining 81 PNGSs, 79 were computationally glycosylated. During the first round, glycosylation of sites N160 (protomer C), N399 (protomer B), and N386 (protomer C) failed. However, after a 30 ns simulation (according to the steps described below), we retried glycosylating these three sites; only site N386 (protomer C) was successfully glycosylated, whereas sites N160 (protomer C) and N399 (protomer B) remained unglycosylated. It is unknown whether, but unlikely that, the three protomers of an Env trimer will always have matching glycosylation profiles; although position N160 is widely known as a conserved PNGS, researchers have used mass spectrometry to quantify the presence of glycans at PNGSs of 94 cross-clade HIV-1 gp120 proteins and the results show that glycosylation at N160 is not absolute^68^. Furthermore, the dependence of a glycan at this position for effective neutralisation has been shown to be variable^12^, and the particular binding characteristics of bNAbs PG9 and PG16 to glycan N160 does not necessitate trimer specific glycosylation, i.e. the antibodies recognise a single N160 glycan^69^. Therefore, although we could not glycosylate each PNGS of the model (positions N335 and N678, and positions N160 on protomer C and N399 protomer B), each viral clone will have varied glycosylation patterns and our ‘fully’ glycosylated model represents one form of the CAP45.G3 Env glycoprotein. We continued with this fully glycosylated model to create the N301A mutant by replacing the asparagine residue at position 301 (HXB2 numbering) with an alanine residue and removing the glycan.

The systems were created using the tLEaP package contained in AmberTools 14^70^. The ff14SB^71^ force field was used for the protein and the GLYCAM06j-1^72^ force field for the glycans. The wild-type and N301A mutant systems were immersed in a truncated octahedron water box containing TIP5P^73^ water molecules, since TIP5P was found to produce the best quantitative agreement with experimental data^74^. The box size was set such that all protein and glycan atoms were 15 Å from the edge of the box. Chloride ions were added to neutralise the system.

#### Simulation

The molecular dynamics simulations were produced using AMBER 14^70^. The systems were minimised by running 10,000 steps of steepest descent and 10,000 steps of conjugate gradient minimisation. During minimisation restraints were placed on all non-hydrogen protein and glycan atoms. The systems were equilibrated by running 0.4 ns simulations under nPT (1 bar, 300 K) on a CPU cluster. During the first equilibration stage, Cartesian restraints (5.0 kcal/mol) were placed on all non-hydrogen protein and glycan atoms. The restraints were removed, and the equilibration was extended by another 1 ns on a GPU cluster to ensure stability across clusters before the production run started on the GPU cluster. The 520 ns production runs were generated on a GPU cluster using AMBER GPU acceleration pmemd^70^ and 0.002 ps time steps, with coordinates written to the trajectory file every 10,000 steps.

### Analyses

#### Root Mean Square Deviation to determine system equilibrium

The conformational stability of the protein during the simulation was assessed by calculating the root mean squared deviation (RMSD) between the protein backbone atoms (C, C-alpha, N, O) and the starting structure of the production run. Conformational stability was achieved after 20 ns (data not shown) and this section of each trajectory was therefore discarded; the remaining 500 ns simulations were used for further analyses.

#### Antibody-accessible surface area for different regions and residues

To investigate the efficiency with which the glycan shield compensates for the loss of glycan N301, we calculated the antibody accessible surface area (AASA) using a 10 Å probe (as an approximation of the size of an antibody^51^) with Naccess^52^ for both the wild-type and N301A mutant simulations. The van der Waals radii of the glycan atoms were defined as described for the GLYCAM06j-1^72^ force field. The AASA was calculated for 2,500 evenly spaced frames across the 500 ns trajectory.

Differences between the wild-type and N301A mutant AASA values could be attributed to either the protein and/or glycan movements and we, therefore, normalise the values to remove changes in the AASA due to protein movements. To do this, we determined the “base/maximum” AASA by removing the glycans and re-calculating the AASA for these non-glycosylated frames. The final AASA ratio, per frame, is the ratio of the glycosylated and non-glycosylated AASA values, calculated by dividing the AASA of the glycosylated protein by the AASA of the non-glycosylated protein. Averages were calculated using these AASA ratio over time.

We compared the mean AASA ratio, per residue, of the N301A mutant simulation to that of the wild-type to determine whether there was a statistically significant increase. The AASA ratio distribution of each residue under the null-hypothesis (wild-type and N301A mutant means are equal) was assessed by using 100 moving-blocks bootstrap replicates. The AASA ratio datasets for each model were mean normalised to satisfy the null-hypothesis before bootstrap replicates were drawn. The 50 ns blocks were defined such that each block could only start on whole nanoseconds for adequate sampling of the whole trajectory. There were, therefore, 450 possible blocks, with ten random blocks required for each bootstrap replicate. Variance normalisation was not performed due to numerous residues with average AASA ratio over time equal to zero. We further filtered the significant residues to include only those where the difference between the average AASA ratio for the wild-type and N301A mutant over time was 10% or greater. We opted for moving-blocks bootstrap above a normal bootstrap approach to conserve the correlation between sequential observations.

#### Nearest glycan calculation for all protein residues

Since the AASA results revealed that some residues have increased AASA ratios in the N301A mutant when compared to the wild-type simulation, we wanted to understand the shielding capacity of the surrounding glycans and determine which glycans are the most likely to affect the AASA ratio of particular protein residues. To achieve this, we calculated, for each residue, the distances between its atoms and all the atoms of the glycans. Each residue is subsequently assigned a nearest glycan based on the calculated distances. The nearest glycan for each residue was calculated for 25 evenly spaced frames across the 500 ns trajectory. The glycan that was most frequently the nearest to a particular residue throughout the trajectory, was defined as that residue’s “nearest glycan”.

#### Overlap between wild-type glycans and specific glycans from the N301A mutant model

The nearest glycan calculation revealed which glycans were nearest to the protein residues that had increased AASA ratios, and showed conformational changes, during the N301A mutant simulation compared to that of the wild-type. In order to determine if these conformational changes were specifically due to the additional space generated from the absence of glycan N301, we investigated whether the changes were viable when glycan N301 was present, i.e. whether or not the identified glycans caused large conformational clashes with any of the wild-type glycans. Hence, we iteratively removed the identified nearest glycans from the wild-type model trajectory and replaced each with the N301A mutant model trajectory equivalent. One glycan was analysed at a time and then restored before the next N301A mutant glycan’s overlap calculations were performed. All the frames of the wild-type and N301A mutant models were aligned to the first frame of the wild-type trajectory before the analysis was carried out. The overlap was calculated using UCSF Chimera^75^ for 2,500 evenly spaced frames across the 500 ns trajectory. The default values were used for the van der Waals overlap (0.6 Å) and potential hydrogen bonding between clashing pairs (subtract 0.4 Å).

#### Tree representation of hydrogen bonds between glycans to illustrate the cascade of events after the loss of a glycan

In order to determine which further glycans were affected by the absence of glycan N301, but not immediately adjacent to the N301A mutation, we calculated and compared the hydrogen bonds that formed between glycans in the N301A mutant and wild-type simulations. The number of hydrogen bonds that formed between any two glycans was calculated for all 25,000 frames. The proportion of the total frames in which there was a hydrogen bond between any two glycans was calculated and rounded to the nearest percentage. The analysis was performed using the hbond function in cpptraj^76^ contained in AmberTools 14, using the default cut-off values for the distance between the heavy atoms (3 Å) and the angle between the acceptor and donor atom (135 degrees). The glycans that showed a change of 10% or greater in the frequency of the interaction between the wild-type and N301A mutant simulations were represented on tree graphs. The analyses tree graphs were generated using R^77^ and, where necessary, the trajectory data was parsed into R using the Bio3D package^78^. Visual representation of the protein structures were generated using UCSF Chimera^75^.

## Electronic supplementary material

Comparison of the CAP45.G3 and Du156.12 molecular dynamics simulations

## Data Availability

The datasets generated during and/or analysed during the current study are available from the corresponding author on reasonable request.

## References

[1] Mascola JR, Haynes BF (2013). HIV-1 neutralizing antibodies: understanding nature’s pathways. Immunol. Rev..

[2] Starcich BR (1986). Identification and characterization of conserved and variable regions in the envelope gene of HTLV-III/LAV, the retrovirus of AIDS. Cell.

[3] Robey WG (1985). Characterization of envelope and core structural gene products of HTLV-III with sera from AIDS patients. Science.

[4] Veronese FD (1985). Characterization of gp41 as the transmembrane protein coded by the HTLV-III/LAV envelope gene. Science.

[5] Moore PL (2008). The c3-v4 region is a major target of autologous neutralizing antibodies in human immunodeficiency virus type 1 subtype C infection. J. Virol..

[6] Richman DD, Wrin T, Little SJ, Petropoulos CJ (2003). Rapid evolution of the neutralizing antibody response to HIV type 1 infection. Proc. Natl. Acad. Sci. USA.

[7] Sagar M, Wu X, Lee S, Overbaugh J (2006). Human immunodeficiency virus type 1 V1-V2 envelope loop sequences expand and add glycosylation sites over the course of infection, and these modifications affect antibody neutralization sensitivity. J. Virol..

[8] Wei X (2003). Antibody neutralization and escape by HIV-1. Nature.

[9] Lasky LA (1986). Neutralization of the AIDS retrovirus by antibodies to a recombinant envelope glycoprotein. Science.

[10] Montagnier L (1985). Identification and antigenicity of the major envelope glycoprotein of lymphadenopathy-associated virus. Virology.

[11] Lynch RM (2015). HIV-1 fitness cost associated with escape from the VRC01 class of CD4 binding site neutralizing antibodies. J. Virol..

[12] Moore PL (2012). Evolution of an HIV glycan-dependent broadly neutralizing antibody epitope through immune escape. Nat. Med..

[13] Moyo T (2017). Chinks in the armor of the HIV-1 Envelope glycan shield: Implications for immune escape from anti-glycan broadly neutralizing antibodies. Virology.

[14] Townsley S, Li Y, Kozyrev Y, Cleveland B, Hu S-L (2015). Conserved Role of an N-Linked Glycan on the Surface Antigen of Human Immunodeficiency Virus Type 1 Modulating Virus Sensitivity to Broadly Neutralizing Antibodies against the Receptor and Coreceptor Binding Sites. J. Virol..

[15] Wang W (2013). A systematic study of the N-glycosylation sites of HIV-1 envelope protein on infectivity and antibody-mediated neutralization. Retrovirology.

[16] Kornfeld R, Kornfeld S (1985). Assembly of asparagine-linked oligosaccharides. Annu. Rev. Biochem..

[17] Wyatt R (1998). The antigenic structure of the HIV gp120 envelope glycoprotein. Nature.

[18] Binley JM (2004). Comprehensive cross-clade neutralization analysis of a panel of anti-human immunodeficiency virus type 1 monoclonal antibodies. J. Virol..

[19] Gao F (2014). Cooperation of B cell lineages in induction of HIV-1-broadly neutralizing antibodies. Cell.

[20] Hraber P (2014). Prevalence of broadly neutralizing antibody responses during chronic HIV-1 infection. AIDS Lond. Engl..

[21] Tomaras GD (2011). Polyclonal B cell responses to conserved neutralization epitopes in a subset of HIV-1-infected individuals. J. Virol..

[22] Wu X (2010). Rational design of envelope identifies broadly neutralizing human monoclonal antibodies to HIV-1. Science.

[23] Muster T (1993). A conserved neutralizing epitope on gp41 of human immunodeficiency virus type 1. J. Virol..

[24] Buchacher A (1994). Generation of human monoclonal antibodies against HIV-1 proteins; electrofusion and Epstein-Barr virus transformation for peripheral blood lymphocyte immortalization. AIDS Res. Hum. Retroviruses.

[25] Walker LM (2009). Broad and potent neutralizing antibodies from an African donor reveal a new HIV-1 vaccine target. Science.

[26] Walker LM (2011). Broad neutralization coverage of HIV by multiple highly potent antibodies. Nature.

[27] Falkowska E (2014). Broadly neutralizing HIV antibodies define a glycan-dependent epitope on the prefusion conformation of gp41 on cleaved envelope trimers. Immunity.

[28] Sather DN (2009). Factors associated with the development of cross-reactive neutralizing antibodies during human immunodeficiency virus type 1 infection. J. Virol..

[29] Wu X (2012). Selection pressure on HIV-1 envelope by broadly neutralizing antibodies to the conserved CD4-binding site. J. Virol..

[30] Sanders Rogier W, van Anken Eelco, Nabatov Alexei A, Liscaljet I Marije, Bontjer Ilja, Eggink Dirk, Melchers Mark, Busser Els, Dankers Martijn M, Groot Fedde, Braakman Ineke, Berkhout Ben, Paxton William A (2008). The carbohydrate at asparagine 386 on HIV-1 gp120 is not essential for protein folding and function but is involved in immune evasion. Retrovirology.

[31] Sok D (2014). Promiscuous glycan site recognition by antibodies to the high-mannose patch of gp120 broadens neutralization of HIV. Sci. Transl. Med..

[32] Travers, S. A. Conservation, compensation, and evolution of N-linked glycans in the HIV-1 group M subtypes and circulating recombinant forms. *ISRN AIDS* **2012**, (2012). 10.5402/2012/823605 PMC3765798 24052884

[33] Koch M (2003). Structure-based, targeted deglycosylation of HIV-1 gp120 and effects on neutralization sensitivity and antibody recognition. Virology.

[34] Li Y (2008). Removal of a single N-linked glycan in human immunodeficiency virus type 1 gp120 results in an enhanced ability to induce neutralizing antibody responses. J. Virol..

[35] Malenbaum SE (2000). The N-terminal V3 loop glycan modulates the interaction of clade A and B human immunodeficiency virus type 1 envelopes with CD4 and chemokine receptors. J. Virol..

[36] McCaffrey RA, Saunders C, Hensel M, Stamatatos L (2004). N-linked glycosylation of the V3 loop and the immunologically silent face of gp120 protects human immunodeficiency virus type 1 SF162 from neutralization by anti-gp120 and anti-gp41 antibodies. J. Virol..

[37] Zolla-Pazner S (2015). Structure/Function Studies Involving the V3 Region of the HIV-1 Envelope Delineate Multiple Factors That Affect Neutralization Sensitivity. J. Virol..

[38] Behrens A-J, Crispin M (2017). Structural principles controlling HIV envelope glycosylation. Curr. Opin. Struct. Biol..

[39] Cutalo JM, Deterding LJ, Tomer KB (2004). Characterization of Glycopeptides From HIV-ISF2 gp120 by Liquid Chromatography Mass Spectrometry. J. Am. Soc. Mass Spectrom..

[40] Go EP (2015). Comparative Analysis of the Glycosylation Profiles of Membrane-Anchored HIV-1 Envelope Glycoprotein Trimers and Soluble gp140. J. Virol..

[41] Go EP, Hua D, Desaire H (2014). Glycosylation and disulfide bond analysis of transiently and stably expressed clade C HIV-1 gp140 trimers in 293T cells identifies disulfide heterogeneity present in both proteins and differences in O-linked glycosylation. J. Proteome Res..

[42] Go EP (2013). Characterization of host-cell line specific glycosylation profiles of early transmitted/founder HIV-1gp120 envelope proteins. J. Proteome Res..

[43] Go EP (2011). Characterization of glycosylation profiles of HIV-1 transmitted/founder envelopes by mass spectrometry. J. Virol..

[44] Go EP (2009). Glycosylation site-specific analysis of clade C HIV-1 envelope proteins. J. Proteome Res..

[45] Go EP (2008). Glycosylation site-specific analysis of HIV envelope proteins (JR-FL and CON-S) reveals major differences in glycosylation site occupancy, glycoform profiles, and antigenic epitopes’ accessibility. J. Proteome Res..

[46] Irungu J (2008). Comparison of HPLC/ESI-FTICR MS versus MALDI-TOF/TOF MS for glycopeptide analysis of a highly glycosylated HIV envelope glycoprotein. J. Am. Soc. Mass Spectrom..

[47] Pabst M, Chang M, Stadlmann J, Altmann F (2012). Glycan profiles of the 27 N-glycosylation sites of the HIV envelope protein CN54gp140. Biol. Chem..

[48] Yang W (2014). Glycoform analysis of recombinant and human immunodeficiency virus envelope protein gp120 via higher energy collisional dissociation and spectral-aligning strategy. Anal. Chem..

[49] Zhu X, Borchers C, Bienstock RJ, Tomer KB (2000). Mass spectrometric characterization of the glycosylation pattern of HIV-gp120 expressed in CHO cells. Biochemistry.

[50] Pritchard LK (2015). Glycan Microheterogeneity at the PGT135 Antibody Recognition Site on HIV-1 gp120 Reveals a Molecular Mechanism for Neutralization Resistance. J. Virol..

[51] Pancera M (2014). Structure and immune recognition of trimeric pre-fusion HIV-1 Env. Nature.

[52] Hubbard, S. & Thornton, J. *NACCESS*. (University College, London, Department of Biochemistry and Moelcular Biology, 1993).

[53] Zhou T (2010). Structural basis for broad and potent neutralization of HIV-1 by antibody VRC01. Science.

[54] Lemmin T, Soto C, Stuckey J, Kwong PD (2017). Microsecond Dynamics and Network Analysis of the HIV-1 SOSIP Env Trimer Reveal Collective Behavior and Conserved Microdomains of the Glycan Shield. Struct. Lond. Engl. 1993.

[55] Dunfee RL (2007). Loss of the N-linked glycosylation site at position 386 in the HIV envelope V4 region enhances macrophage tropism and is associated with dementia. Virology.

[56] Yang M, Huang J, Simon R, Wang L-X, MacKerell AD (2017). Conformational Heterogeneity of the HIV Envelope Glycan Shield. Sci. Rep..

[57] Zhou T (2017). Quantification of the Impact of the HIV-1-Glycan Shield on Antibody Elicitation. Cell Rep..

[58] Stewart-Jones G (2016). Trimeric HIV-1-Env structures define glycan shields from clades A, B, and G. Cell.

[59] Ward AB, Wilson IA (2017). The HIV-1 Envelope Glycoprotein Structure: Nailing down a Moving Target. Immunol. Rev..

[60] Lyumkis D (2013). Cryo-EM structure of a fully glycosylated soluble cleaved HIV-1 envelope trimer. Science.

[61] Sali A, Blundell TL (1993). Comparative protein modelling by satisfaction of spatial restraints. J. Mol. Biol..

[62] Webb B, Sali A (2014). Comparative protein structure modeling using MODELLER. Curr. Protoc. Bioinforma..

[63] Julien J-P (2013). Crystal structure of a soluble cleaved HIV-1 envelope trimer. Science.

[64] Huang C (2005). Structure of a V3-containing HIV-1 gp120 core. Science.

[65] Shen M-Y, Sali A (2006). Statistical potential for assessment and prediction of protein structures. Protein Sci. Publ. Protein Soc..

[66] GLYCAM. Available at: http://glycam.org/. (Accessed: 10th January 2018).

[67] Petrescu A, Milac A, Petrescu S, Dwek R, Wormald M (2004). Statistical analysis of the protein environment of N-glycosylation sites: implications for occupancy, structure, and folding. Glycobiology.

[68] Yu Wen-Han, Zhao Peng, Draghi Monia, Arevalo Claudia, Karsten Christina B., Suscovich Todd J., Gunn Bronwyn, Streeck Hendrik, Brass Abraham L., Tiemeyer Michael, Seaman Michael, Mascola John R., Wells Lance, Lauffenburger Douglas A., Alter Galit (2018). Exploiting glycan topography for computational design of Env glycoprotein antigenicity. PLOS Computational Biology.

[69] McLellan JS (2011). Structure of HIV-1 gp120 V1/V2 domain with broadly neutralizing antibody PG9. Nature.

[70] Case, D. *et al*. *AMBER 2014*. (University of California, 2014).

[71] Maier JA (2015). ff14SB: Improving the Accuracy of Protein Side Chain and Backbone Parameters from ff99SB. J. Chem. Theory Comput..

[72] Kirschner KN (2008). GLYCAM06: a generalizable biomolecular force field. Carbohydrates. J. Comput. Chem..

[73] Mahoney MW, Jorgensen WL (2000). A five-site model for liquid water and the reproduction of the density anomaly by rigid, nonpolarizable potential functions. J. Chem. Phys..

[74] Sauter J, Grafmüller A (2015). Solution properties of hemicellulose polysaccharides with four common carbohydrate force fields. J. Chem. Theory Comput..

[75] Pettersen EF (2004). UCSF Chimera–a visualization system for exploratory research and analysis. J. Comput. Chem..

[76] Roe DR, Cheatham TE (2013). PTRAJ and CPPTRAJ: Software for Processing and Analysis of Molecular Dynamics Trajectory Data. J. Chem. Theory Comput..

[77] R Core Team. *R: A Language and Environment for Statistical Computing*. (R Foundation for Statistical Computing, 2016).

[78] Grant BJ, Rodrigues APC, ElSawy KM, McCammon JA, Caves LSD (2006). Bio3d: an R package for the comparative analysis of protein structures. Bioinforma. Oxf. Engl..

