# Supplementary material for: Structural Rearrangements Maintain the Glycan Shield of an HIV-1 Envelope Trimer After the Loss of a Glycan
Source: Sci Rep. 2018 Oct 9;8:15031. doi: 10.1038/s41598-018-33390-2 (PMC6177452; doi:10.1038/s41598-018-33390-2)
Supplement: Supplementary file 1 — Comparison of the CAP45.G3 and Du156.12 molecular dynamics simulations [file 41598_2018_33390_MOESM1_ESM.docx]

**Supplementary File 1**

**Structural Rearrangements Maintain the Glycan Shield of an HIV-1 Envelope Trimer After the Loss of a Glycan**

Roux-Cil Ferreira, Oliver C. Grant, Thandeka Moyo, Jeffrey R. Dorfman, Robert J. Woods, Simon A. Travers, Natasha T. Wood

**Comparison of the CAP45.G3 and Du156.12 molecular dynamics simulations**

Our preliminary analysis was carried out on the two homology models generated using the HIV-1 Env sequences from two isolates previously reported on in Moyo *et al*. Briefly, during the in vitro study of the two subtype C strains, which share approximately 90% sequence identity, the glycan shield was remodelled and tested against a panel of sera ($n$=64) from chronically HIV-1 infected individuals. This was done to assess whether the epitopes of frequently elicited neutralising antibodies were exposed on the remodelled shield. It was shown that the Du156.12 strain required glycan N301 for maintenance of its glycan shield, whereas the CAP45.G3 strain was, comparatively, less reliant on glycan N301, since it remained resistant to the panel of sera despite the loss of this glycan.

The initial state minimisation molecular modelling carried out in Moyo *et al.*, revealed a notable increase in [solvent accessible surface area](https://www.sciencedirect.com/topics/medicine-and-dentistry/accessible-surface-area) upon removal of the glycan at position 301 for the Du156.12 models when compared to the CAP45.G3 models. Therefore, the preliminary structural analysis suggested that glycan N301 blocked a larger surface area blocked on the Du156.12 homology structure than on the CAP45.G3 structures.

We followed the analysis from the initial study by carrying out molecular dynamics simulations (500ns each) on the CAP45.G3 and Du156.12 wild-type, and corresponding N301A mutant, structures. We then aimed to extend the [solvent accessible surface area](https://www.sciencedirect.com/topics/medicine-and-dentistry/accessible-surface-area) calculation from the Moyo *et al.* study, which was carried out on 10 homology models, to 276 snapshots from the molecular dynamics simulations. For each snapshot, and each residue on the model, we calculated the [solvent accessible surface area](https://www.sciencedirect.com/topics/medicine-and-dentistry/accessible-surface-area) using several increasing probe sizes (1.4 Å, 2 Å, 4 Å, 6 Å, 8 Å, 10 Å). For example, if a residue was accessible by a probe of size 8 Å at the first time point, but not by a probe of size 10 Å, the accessible surface area at that point was noted as 8 Å. This was repeated for each snapshot/time point. The average accessible surface area was then determined and this value was depicted on the trimer models (Supplementary Figure 1). We then specifically focussed on the differences in accessibility of the V3-loop and CD4 binding site regions, which are targeted by the anti-V3 and anti-CD4 antibodies, and observed that the residues of these regions on the CAP45.3G N301A mutant model, appeared, on average, more accessible (more blue, Supplementary Figure 1) than the corresponding residues on the Du156.12 N301A mutant models (Supplementary Figure 1). Only for protomer B did the accessibility appear swapped.


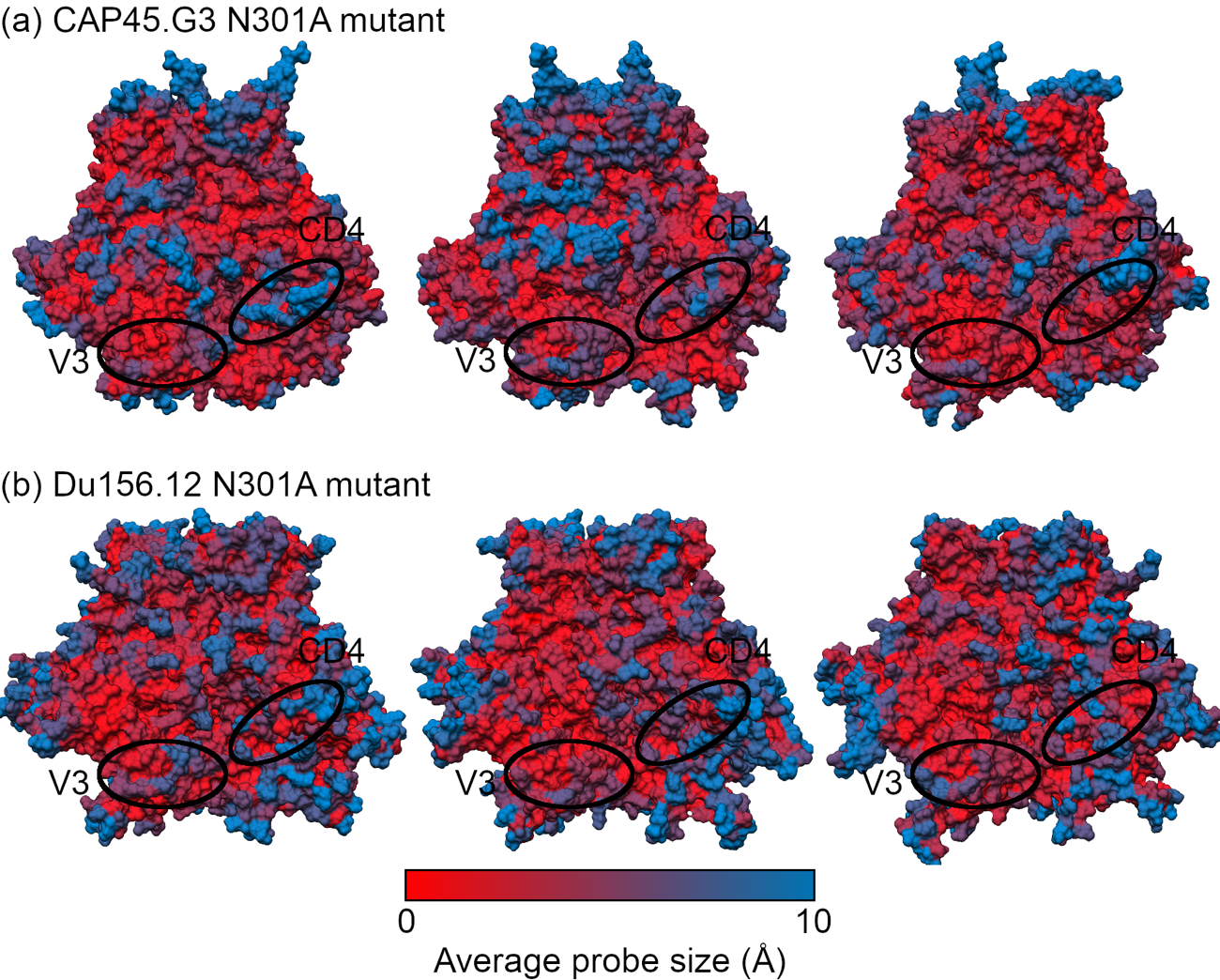


**Supplementary Figure 1.** Residue accessibility for the CAP45.G3 and Du156.12 N301A mutant models.

The surface area accessibility for each residue on the (a) CAP45.G3 and (b) Du156.12 N301A mutant models is shown on a scale where bright red represents least accessible and blue indicates high accessibility. The V3-loop and CD4 binding site regions are circled and labelled.

To quantify the visual differences, we calculated the average accessible surface area for the V3-loop or CD4 binding site regions, as well as for the residues that form part of the VRC01 epitope. We found that for protomer A and C, the three regions were less accessible on the CAP45.G3 N301A mutant model than for the Du156.12 N301A mutant model (Supplementary Table 1).

**Supplementary Table 1.** Average accessible surface area (in Å) for specific regions of each protomer of the CAP45.G3 and Du156.12 N301A mutant models

|  | **Protomer** | **V3-loop region** | **CD4 binding site region** | **VRC01 epitope** |
| --- | --- | --- | --- | --- |
| **CAP45.G3** | A | 3.202816 | 3.663491 | 4.089400 |
|  | B | 3.607805 | 4.824488 | 5.366646 |
|  | C | 2.965114 | 3.913406 | 4.236977 |
|  |  |  |  |  |
| **Du156.12** | A | 3.454886 | 4.450575 | 5.040000 |
|  | B | 3.515880 | 3.961275 | 4.664886 |
|  | C | 3.002402 | 4.455136 | 5.175756 |

This comparison suggested that the surface of residues that form part of the VRC01 antibody epitope, CD4 binding site region and V3-loop region were, on average and across protomers, more exposed on the Du156.12 N301A mutant model than on the CAP45.G3 N301A mutant model. However, during the initial comparison we also noted that the distribution of glycans on the two models was substantially different and that these differences make direct comparison between the models difficult.

Thus, since the key observation during our initial, *in silico*, comparative analysis was that the conformational differences of the glycans on the wild-type models, as well as the landscapes around each of these glycans, likely affected the ultimate changes in the glycan shields when the N301 glycan was removed, we shifted our focussed to the collective glycan behaviour observed on one model. Since Moyo *et al.* speculated that the CAP45.G3 virus typified a subset of viruses where the loss of glycan N301 was tolerated, i.e. the protective qualities of the glycan shield, or perhaps the glycan microdomain, were retained, the focus of our manuscript was on providing a thorough account for the suggested compensation of this virus’ glycan shield.

**References:**

Moyo, T. et al. Chinks in the armor of the HIV-1 Envelope glycan shield: Implications for immune escape from anti-glycan broadly neutralizing antibodies. Virology 501, 12–24 (2017).

Lemmin, T., Soto, C., Stuckey, J. & Kwong, P. D. Microsecond Dynamics and Network Analysis of the HIV-1 SOSIP Env Trimer Reveal Collective Behavior and Conserved Microdomains of the Glycan Shield. Struct. Lond. Engl. 1993 25, 1631-1639.e2 (2017).
